# Halitosis: the unique scent of colorectal cancer

**DOI:** 10.17179/2025-8338

**Published:** 2025-05-26

**Authors:** Ying Chen, Xiao Xian Qian

**Affiliations:** 1 Department of Gastroenterology and Halitosis Clinic, Minhang Hospital, Fudan University, Shanghai, China

## ⁯⁯⁯

We read with great interest the newly published article by Alsaadi et al. (2024[1]). In the article, they conducted a systematic appraisal and meta-analysis of the published evidence searched from nine electronic databases until August 2020 to determine whether exhaled volatile organic compounds (VOCs) can be used in the detection and screening of colorectal cancer (CRC) (Alsaadi et al., 2024[1]).

As gastroenterologists and halitosis professionals, we have noted for years that many CRC patients, who have no notable intra-oral conditions, can also experience halitosis of varying degrees. This halitosis has a complex and distinct scent somewhat similar to rotten meat, yet it is difficult to describe precisely. Halitosis, also known as bad breath or oral malodor, refers to the offensive malodor beyond a socially acceptable level emitted from a subject's mouth and/or nose (Xu et al., 2024[10]). Intra-oral conditions such as periodontitis and coated tongue, and extra-oral conditions such as respiratory tract infections and liver cirrhosis, can consequently lead to intra-oral halitosis and extra-oral halitosis, respectively. A variety of odorants, mainly comprising volatile sulfur compounds (VSCs) and VOCs, contribute to the halitosis perceived in patients (Xu et al., 2024[10]). 

The distinct scent in breath of CRC patients has also been noted by Sonoda et al. (2011[8]). They employed a Labrador retriever specially trained in scent detection of cancer. The dog first smelled a standard breath sample of a CRC patient, and then smelled other breath samples while cooperating with a handler. The researchers found that, among patients with CRC and controls, the sensitivity of canine scent detection of breath samples compared with conventional diagnosis by colonoscopy was 0.91 and the specificity was 0.99. This suggests that a specific cancer scent does indeed exist in the breath of CRC patients, and cancer-specific chemical compounds may become effective tools in CRC screening. Accordingly, to identify those CRC-specific chemical compounds, we have conducted breath tests using the portable gas chromatography (GC) OralChroma (CHM-1, Abimedical, Osaka, Japan) on dozens of these CRC patients with halitosis in a consultation room. The device is designed to specifically measure three major VSCs-hydrogen sulfide (H_2_S, H-S-H), methyl mercaptan (MM, CH_3_-SH), and dimethyl sulfide (DMS, CH_3_-S-CH_3_). We have discovered significantly elevated dimethyl sulfide in their breath, while low levels of hydrogen sulfide or methyl mercaptan were detected. However, according to previous reports, dimethyl sulfide has a disagreeable odor resembling wild radish or cabbage (Harvey-Woodworth, 2013[3]), or an unpleasantly sweet odor (Tangerman and Winkel, 2010[9]). Therefore, dimethyl sulfide alone could not explain the complex halitosis of these CRC patients, which has puzzled us for years.

Drawing inspiration from the work of Alsaadi et al. (2024[1]), to find more clues regarding the potential odorants contributing to this complex halitosis, we have searched in PubMed and carefully examined published original articles focusing on the breath VOCs profiles of CRC patients by March 10, 2025, especially those studies that compared CRC patients with controls. As a result, we have obtained some meaningful findings. For instance, Amal et al. (2016[2]) conducted a breath analysis using gas chromatography-mass spectrometry (GC-MS), and found higher level of acetone in CRC patients compared to controls. Similarly, Śmiełowska et al. (2023[7]) conducted a breath analysis using thermal desorption-gas chromatography-mass spectrometry (TD-GC-MS), and found elevated amounts of acetone and heptanoic acid in CRC patients compared to healthy volunteers. In contrast, Kononova et al. (2024[4]) conducted a breath analysis using GC-MS, and found 2-butanone at a statistically significantly higher concentration in the CRC group compared to controls. Meanwhile, Markar et al. (2019[6]) conducted a breath analysis using selected-ion-flow-tube MS, and found that propanal was significantly elevated in the CRC cohort compared with controls. Liu et al. (2024[5]) conducted a breath analysis using GC-MS/MS, and found that allyl methyl sulfide (AMS, C_4_H_8_S), a volatile organic sulfur compound (VOSC), was significantly elevated in the CRC cohort compared with healthy controls. According to PubChem (https://pubchem.ncbi.nlm.nih.gov/) and previous reports, acetone has a characteristic sweetish but pungent odor; heptanoic acid has a disagreeable rancid odor; 2-butanone has a nauseating odor; propanal has an overpowering fruity-like but pungent unpleasant, choking odor; and AMS has a strong offensive odor of onions or garlic (Tangerman and Winkel, 2010[9]). 

Accordingly, we postulate that the elevated dimethyl sulfide, acetone, heptanoic acid, 2-butanone, propanal and allyl methyl sulfide previously discovered by us and other scholars, may constitute the current odorant panel contributing to the complex halitosis of CRC patients. Therefore, halitosis can potentially be regarded as the unique scent of CRC. A promising application is the future development of a portable GC instrument, specially designed and similar to OralChroma, that can specifically detect these six odorants to achieve a more feasible and cost-effective strategy for CRC screening.

Lastly, we would like to express our utmost gratitude to Alsaadi et al. for their work, as it has inspired us to do the work described above and provided guidance for our future exploration of VOCs related to halitosis in CRC patients.

## Declaration

### Acknowledgments

None.

### Author contributions

Ying Chen - conceptualization and writing. Xiao Xian Qian - conceptualization, data collection and analysis, writing, supervision and reviewing.

### Data Availability

No data were generated in this study.

### Funding

This study was funded by the High Level Specialized Backbone Physician Training Project (2024-2027) in the Medicine, Teaching and Research Collaborative Health Service System of Minhang District granted to Xiao Xian Qian (grant number: 2024MZYS003)..

### Conflict of interest

The authors declare that they have no conflict of interest.

### Ethical approval and consent to participate

Not applicable.

### Using artificial intelligence (AI)

The authors disclose that they have not used AI-assisted technologies (such as Large Language Models [LLMs], chatbots, or image creators) in the production of submitted work.
